# Examining gender bias in regional anesthesia academic publishing: a 50-year bibliometric analysis

**DOI:** 10.1186/s44158-023-00137-z

**Published:** 2023-12-06

**Authors:** Sindi Mustaj, Alessandro De Cassai, Gaya Spolverato, Tommaso Pettenuzzo, Annalisa Boscolo, Paolo Navalesi, Marina Munari

**Affiliations:** 1 Department of Medicine-DIMED, University of Padua, Padua, Italy; 2 Sant’Antonio Anesthesia and Intensive Care Unit, University Hospital of Padua, Padua, Italy; 3 Department of Surgical, Oncological and Gastroenterological Sciences, University of Padua, Padua, Italy; 4 Anesthesia and Intensive Care Unit, University Hospital of Padua, Padua, Italy; 5 Thoracic Surgery and Lung Transplant Unit, Department of Cardiac, Thoracic, Vascular Sciences, and Public Health, University of Padua, Padua, Italy

**Keywords:** Gender, Equity, Regional anesthesia

## Abstract

**Background:**

The connection between academic career advancement and publishing research articles is important, as it can impact promotion and compensation decisions. Gender bias in academic publishing is a known issue, with studies showing low numbers in key roles in female representation. This article aims to analyze the ratio of women to men as first and last authors in the *Regional Anesthesia & Pain Medicine* (RAPM) journal and explore other factors such as the mentorship effect and representation in regional anesthesia associations.

**Main body:**

We examined the RAPM articles from 1976 to 2023 evaluating the gender of first and last authors. We analyzed the trend over the years and also analyze the subset of original articles. A further analysis was conducted to analyze the relationship between the first and last author’s gender. Additionally, regional anesthesia societies were contacted to gather data on the gender of their members. We included 5650 articles; most of them were first authored by men (72.9–87.7%). There was a positive trend over time for female first authorship but not for last authorship. The analysis also revealed a mentorship effect in recent years for both overall articles and the subgroup of original articles. The representation of women within regional anesthesia societies contrasted with the representation of women as last authors in original articles.

**Conclusions:**

Our findings raise important questions about gender bias in academic publishing highlighting the need for increased representation and opportunities for women in the field of regional anesthesia.

**Supplementary Information:**

The online version contains supplementary material available at 10.1186/s44158-023-00137-z.

## Introduction

Academic career advancement and publishing original research articles go hand in hand, as the publication of articles together with other metrics helps establish competence and can ultimately influence academic promotion [1]. Moreover, decisions on compensation and allocation of nonclinical time are often influenced by an investigator’s scholarly articles, both in terms of their quality and quantity [2, 3].

In this framework, the significance of ensuring equal opportunities for researchers, particularly during the early stages of their career, becomes evident as a core component in fostering academic equity across promotion, compensation, and career advancement. Regrettably, academic gender bias is an established issue in academic publishing [4].

In recent years, numerous studies have explored the role and presence of women in academic publishing, and these investigations have consistently shown a positive trend, highlighting an increase in the representation of women over time [5, 6]. However, the representation of female researchers in the key roles of publishing, namely first and last authors, remains too low as shown by an article published in the *British Journal of Anesthesia* with a 30% of female first author and only a 7% of papers with both a female first and last author [7]. However, specific data on gender gap in regional anesthesia publishing are still lacking; for this reason, we decided to focus our attention on *Regional Anesthesia & Pain Medicine* (RAPM) publication history. RAPM is the official publication of ESRA (European Society of Regional Anesthesia), ASRA (the American Society of Regional Anesthesia and Pain Medicine), AOSRA (the Asian and Oceanic Society of Regional Anesthesia), LASRA (the Latin American Society of Regional Anesthesia), and AFSRA (the African Society for Regional Anesthesia) and is specifically dedicated to regional anesthesia and pain medicine procedures.

The main objective of our research article was to analyze the ratio of women to men in terms of scholarly productivity as first and last authors in RAPM, with the hypothesis that there would be a significant upward trend over the years.

As secondary objectives, we sought to examine whether the demonstrated trend would still hold true when analyzing only the original articles data subset. Furthermore, we aimed to determine if there is a gender mentorship effect, namely the likelihood of the first author’s gender being influenced by the gender of the last author. Lastly, we aimed to assess whether the proportion of female authorship is consistent with the proportion of women membership in associations primarily focused on regional anesthesia (ESRA, ASRA, AOSRA, LASRA, AFSRA).

## Methods

### RAPM authors

We accessed the table of contents of each issue, with the exclusion of special issues of RAPM from 1976 (Volume 1, Issue 1) to 2023 (Volume 48, Issue 8) from the journal archive available on the website (https://rapm.bmj.com/content/by/year).

We retrieved a comprehensive list of all the articles for each issue, excluding articles not reporting the authors in the table of contents. From this list, we extracted the article name, publishing year, manuscript type, number of authors, and the full names of both the first and last authors.

In the case of a manuscript with a single author, we deemed it suitable to designate the author as the first author. In order to ensure the robustness of our analysis, we performed sensitivity analysis by excluding papers with a single author.

To predict the gender of a person given their first name, we used the web service genderize.io (https://genderize.io/); this tool uses machine learning algorithms to estimate the likelihood of a name belonging to a particular gender; using such tool, we were able to associate to each first name the following labels: “male,” “female,” and “unknown” with the associated probability.

### Original article

Initially, our strategy was to classify the original articles by utilizing the labels provided in the table of contents. Yet, during the extraction phase, we came across certain articles that, despite having disparate labels in the table of contents (for example, “obstetric analgesia” and “pediatric analgesia”), were, in fact, original articles. Hence, we decided to undertake a meticulous manual review of all articles to construct an exhaustive compilation of the original content published in the journal.

### Mentorship

Mentorship was defined as the relation between the first and the last author (mentor). In order to examine the impact of gender mentorship, we formed a specific subset from the original data consisting of articles (a) co-authored by at least two individuals, (b) with retrieved gender information for both the first and last authors.

A further subset of data with only original articles was created to perform a sensitivity analysis. Moreover, to offer a comprehensive understanding of this phenomenon over time, we opted also to categorize the timeline into decades on an arbitrary basis.

### Women membership in regional anesthesia societies

In order to examine possible differences among female authorship and proportion of women in regional anesthesia societies, we reached out to each of the identified associations (ESRA, ASRA, AOSRA, LASRA, AFSRA) via mail, requesting yearly data on the gender of their society members dating back to 1976. In instances where we did not receive a response, we followed up with a maximum of three reminders.

### Composition of the editorial board

In order to examine possible impact of editorial board composition on female authorship, we asked to the editor in chief of the journal of interest to provide the composition of the journal over the years

### Statistical analysis

Categorical variables presented were expressed as number and percentages; the comparisons for these variables were performed using the chi-square test or Fisher’s exact test when appropriate.

The Spearman correlation test was employed to assess the existence and the strength of a correlation.

All statistical analyses were conducted using R version 3.4.0 (2017-04-21); statistical significance was set at *p*-values < 0.05.

## Results

We have identified a total of 5650 articles that were published within the considered time frame. Of them, 1133 were authored by a single author, while the remaining 4517 were authored by two or more authors.

Manuscripts with more than one author had as first authors 3292 men (72.9%), 872 women (19.3%), and in 353 cases, it was not possible to determine the author gender (7.8%). A similar pattern was present when considering last authors with 3468 men (76.8%), 702 women (15.5%), and 347 not determined gender (7.7%).

Manuscripts authored by a single author had a woman author in 120 articles (10.6%), a male author in 994 articles (87.7%), and with the remaining unidentified (19, 1.7%).

Based on the information provided, we identified a total of 719 authors whose gender could not be determined. The absence of a complete first name, often represented by initials, resulted in 275 cases of unidentified first authorship, 270 cases of unidentified last authorship, and 68 cases of unidentified single authorship. In all remaining cases, we encountered difficulty in determining the gender, even with the inclusion of a reported first name. This was due to the probability in the database being lower than 0.6 or the name not being found in the database.

### Original article subgroup

We identified a total of 2084 original articles. Of them, only 57 articles were authored by a single author with 43 out of 57 (75.4%) published before 2000.

Manuscripts with more than one author had as first authors 1395 men (68.8%), 427 women (21.1%), and in 205 cases, it was not possible to determine the author gender (10.1%). A similar pattern was present when considering last authors with 1478 men (72.9%), 328 women (16.2%), and 221 not determined gender (10.9%).

Manuscripts authored by a sole author had a woman author in 8 articles (14.3%), a male author in 46 articles (80.7%), and with the remaining unidentified.

### Trends over time

**Fig. 1:**
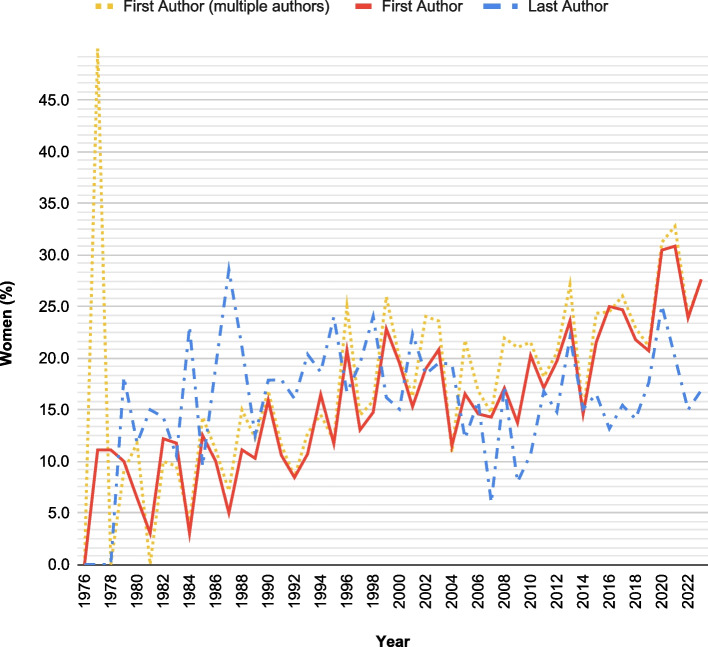
Trend over time of female representation in RAPM articles since 1976

Figure 1 provides a visual representation of the changing trend in female first and last authors over time, taking into account articles with both sole and multiple authors.

Notably, the percentage of women as first authors peaked at 41.0% in 2020, while for last authors, the highest percentage of female representation was recorded in 1995 with 35.0%.

It is notable that over the years, women reached 11 times percentages over 30% for first authorship, and that 9 of these times happened in the last 15 years; however, women as last authors reached such percentage on a single occasion.

The relationship between the proportion of first female authorship and the publishing years exhibits a consistent positive correlation across various aspects, including overall (rho: 0.726, *p*-value: < 0.001) or only multiple-authored (rho: 0.848; *p*-value: < 0.001) manuscripts, as well as when considering all articles or focusing solely on the original articles subgroup (single authored: rho 0.774; *p*-value < 0.001; multiple authored rho 0.760; *p*-value < 0.001).

**Fig. 2:**
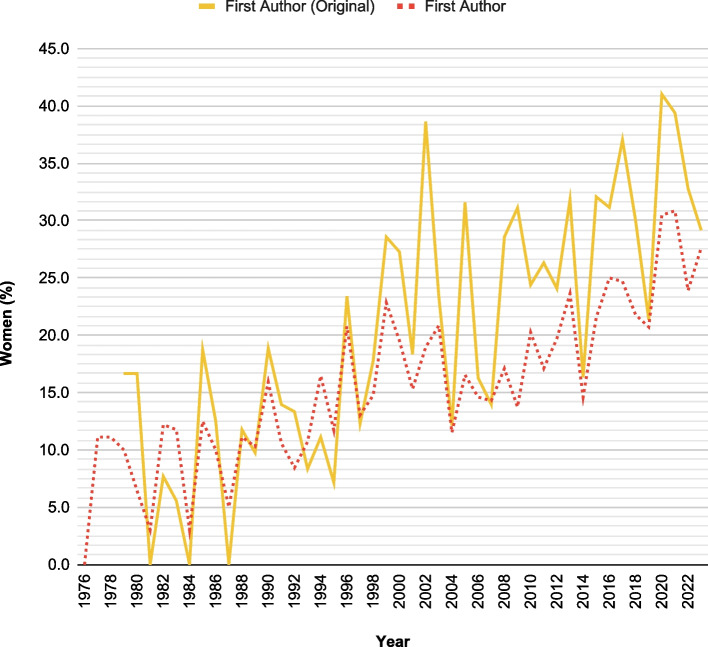
Comparison of female representation as first authors between overall articles and original articles over time

**Fig. 3:**
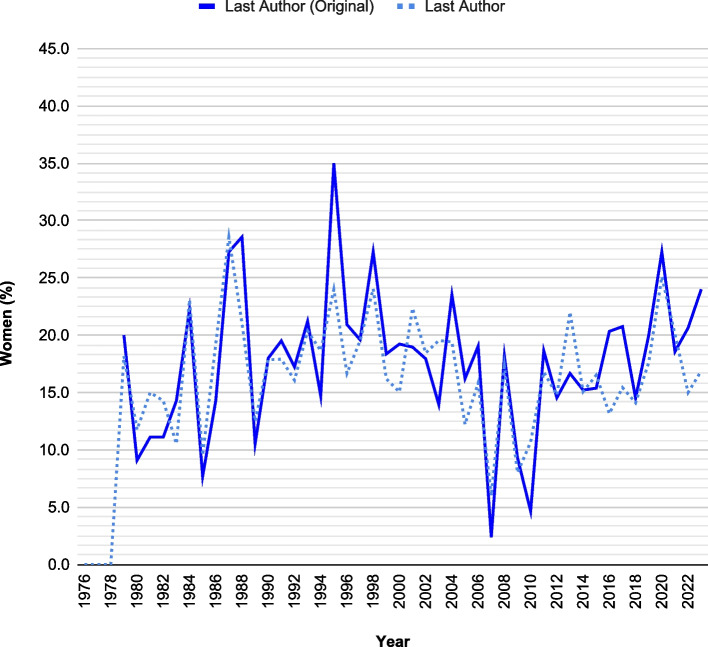
Comparison of female representation as last authors between overall articles and original articles over time

On the contrary, there was no apparent correlation between the gender of the last author and the publishing years, both when considering all articles (rho 0.132, *p*-value 0.369) or only the original articles (rho 0.145; *p*-value 0.340). A graphical comparison of gender representation in all manuscripts and only in the subset of original articles is depicted in Fig. 2 for first authoring and in Fig. 3 for last authoring.

### Mentorship

The subsetting of the data resulted in a database containing a total of 4069 articles (1771 original articles) in this analysis. Results of the analysis, summarized in Table 1, showed a mentorship effect in the most recent years for both the overall articles and the original articles subgroup.

**Table 1:** Proportion of female first authorship according to the gender of the last author

**Time**	**Last author gender**		***p*-value**
**All articles**			
	Men	Women	
Overall	696 (20.5%)	152 (22.3%)	0.307
< 1990	21 (10.1%)	3 (7.9%)	0.668
1990–1999	98 (16.6%)	22 (15.6%)	0.765
2000–2009	154 (18.5%)	31 (21.4%)	0.412
2010–2019	266 (22.8%)	39 (17.8%)	0.101
> 2019	157 (26.5%)	57 (41.0%)	< 0.001*
**Original articles**			
Overall	332 (22.9)	79 (24.6)	0.510
< 1990	12 (8.8)	2 (8.0)	0.893
1990–1999	49 (15.3)	14 (16.1)	0.850
2000–2009	83 (23.6)	18 (27.3)	0.520
2010–2019	131 (28.2)	20 (21.1)	0.154
> 2019	57 (32.4)	25 (52.1%)	0.012*

^*^Statistically significant

### Proportion of women membership in regional anesthesia societies

We received answers from ASRA, ESRA, and AOSRA, while we received no answers from LASRA and ASFRA.

ASRA provided data from 2019 to 2022, ESRA provided data from 2008 to 2023, while AOSRA provided no data (supplementary digital content 1).

**Fig. 4:**
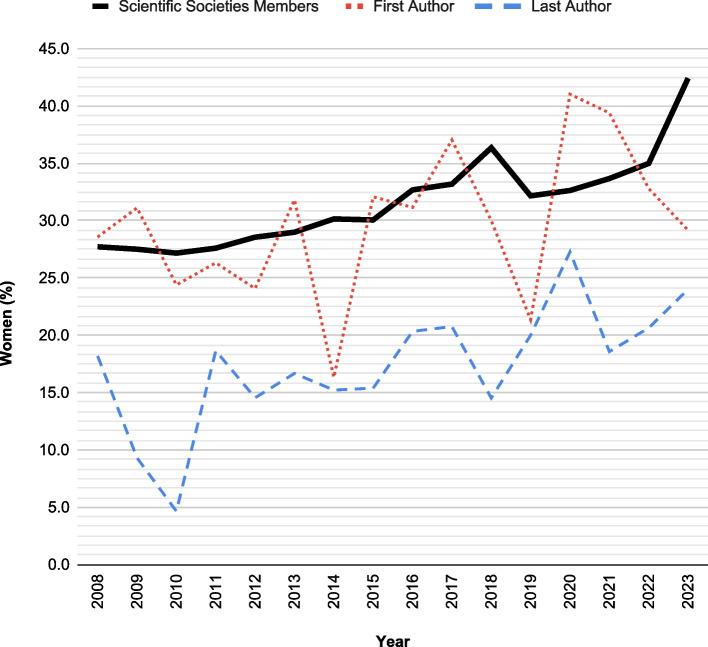
Comparison of female representation in scientific societies and as first and last authors of original articles

A striking contrast is observed when graphically depicting the data comparing the last authorship of original articles with the representation of women within regional anesthesia societies (Fig. 4), exposing a considerable discrepancy in the representation of women within regional anesthesia societies. Conversely, the proportion of women in first authorship aligns with the membership composition of these societies (statistical analysis for this outcome is available as supplementary digital content 2)

### Editorial board composition

The editor in chief promptly addressed our query and quickly connected us with the person in charge at the publisher. However, due to the publisher’s recent adoption of voluntary data tracking, we were unable to obtain the necessary information over the years.

## Discussion

Our research article reveals a notable upward trend in female authorship for the first but not the last author in RAPM over 50 years. These findings remain consistent across both original and non-original articles as well as single- or multiple-authored manuscripts.

Increase in first authorship aligns with another research article that examined five prominent high-impact journals (*Anesthesiology*, *British Journal of Anaesthesia*, *Anaesthesia*, *European Journal of Anaesthesiology*, and *Anesthesia & Analgesia*) reporting an overall increase of 9.4% in female first authorship over a 10-year period [8].

Our article shows that the percentage of female first authors in 2023 was 27.6%, which raises an intriguing question about the presence of gender bias since the percentage falls below 50%.

Based on the analysis of data provided by scientific societies, it is evident that the representation of women members in regional anesthesia societies is in line with the proportion of women who are first authors on papers in the same field.

However, we acknowledge that our data may have some inherent biases. Firstly, we were unable to collect data from AOSRA, LASRA, and ASFRA, which limits the comprehensive nature of our findings. Additionally, there was a proportion of not reported gender in the provided data, further contributing to potential biases.

Nevertheless, data regarding regional anesthesia societies align with a 2019 report by Bissing et al. [9], which also investigated the status of women in academic anesthesiology. According to the report, women anesthesiology residents accounted for 34% of the total in 2016; such percentages are similar to the percentages retrieved in our study.

Last authoring in our study accounted for 16.9% in 2023, with an average value of 15.5% over the years without an apparent increasing or decreasing trend.

The missed increase in last authoring was noted also in a research regarding the *Journal of Cardiothoracic and Vascular Anesthesi*a where female first author increased by 16.6% (9.6% in 1990 versus 26.2% in 2017), while last author increased only by 4.8% (7.0% in 1990 versus 11.8% in 2017) [5].

However, other studies analyzed female last authoring in different journals retrieving similar proportions, for example, 22% in 2017 (*Canadian Journal of Anesthesia* 22%) [10], 20.1% in 2020 (*Journal of Clinical Anesthesia*, *British Journal of Anesthesia*, and *Anesthesiology*) [11].

We believe that the reason for the lower percentage of female last authors could be researched in the similar low proportions of anesthesiology professors. In fact, it was reported that in 2019, women accounted for only 17% of professors in anesthesiology [9], and even if the odds of female professorship in anesthesiology showed a promising increase of 2.9% per 5-year interval ^9^, however, these odds were lower if compared to the odds of any other clinical department (3.9%) [9].

The limited increase in the number of women as last author during the years could represent a barrier to women mentorship. Given the above, we believe that it is crucial to incentivize an increased access to professorships for women, as our research has revealed the potential for a significant mentorship effect. Such an effect (visible in the last years of our analysis) could effectively further reduce gender bias in academic publishing.

A mentor is a key role in the development of a young researcher as it provides support, challenge, and vision making [12] with each of these being crucial for a researcher at the start of the career. However, it is crucial to recognize that sponsorship is equally significant as mentorship. While a mentor provides guidance and support to help a researcher grow and develop over the long term, a sponsor uses their professional connections and influence to actively promote and advocate for the researcher’s career advancement [13]. Both roles are important, as mentorship helps researchers build skills and knowledge, while sponsorship provides the necessary opportunities and exposure to advance their careers. As noted by Rubulotta, strong networking can be a game-changer in terms of career success, making sponsorship a crucial aspect of professional development [14].

An increased access to mentorship and increasing sponsorship possibilities for women could be the game-changer in gender equity.

One potential area for improvement could be to explore the impact of editorial board composition on authorship in the field of regional anesthesia, while many publishers have started addressing the issue of gender imbalance in their editorial boards in recent years, for example, by disclosing their editorial board gender composition on their websites on a volunteer basis. However, several studies have highlighted the need for further action in this regard [15, 16]. It would be fascinating to examine the relationship between editorial board composition and authorship patterns in regional anesthesia in future studies, as this aspect remains largely unknown at present.

Our study has some limitations that warrants discussion.

First, we utilized the genderize.io application to determine gender, but we are aware that alternative applications or strategies could yield different outcomes. Second, our investigation focused solely on authors in the RAPM journal, overlooking other scholarly publications within the field of regional anesthesia. Nevertheless, RAPM is widely regarded as the leading journal in this domain, and a comprehensive trend analysis spanning 50 years provides valuable insights.

Third, it would have been valuable to gather data on the gender composition of the editorial board of RAPM throughout the years. This information could have provided additional insights to supplement our findings on authorship percentages.

Thirdly, we recognize the intricate nature of gender bias in academic publishing and acknowledge that it cannot be solely attributed to the order of publishing. Nonetheless, we believe that our manuscript contributes an important piece to the overall puzzle on the topic.

## Conclusions

In conclusion, our research highlights a significant increase in female first authorship in RAPM but a stagnant trend in female last authorship. These findings raise important questions about gender bias in academic publishing and emphasize the need for increased representation and opportunities for women in the field of regional anesthesia.

### Supplementary Information

**Additional file 1: Supplementary material 1.** Scientific societies provided data.

**Additional file 2: Supplementary material 2.** Comparison  first/last authors and scientific societies composition.

## Data Availability

Dataset is available on reasonable request.

## Abbreviations

RAPM: Regional Anesthesia & Pain Medicine
ESRA: European Society of Regional Anesthesia
ASRA: American Society of Regional Anesthesia and Pain Medicine
AOSRA: Asian and Oceanic Society of Regional Anesthesia
LASRA: Latin American Society of Regional Anesthesia
AFSRA: African Society for Regional Anesthesia
