# Supplementary material for: Examining gender bias in regional anesthesia academic publishing: a 50-year bibliometric analysis
Source: J Anesth Analg Crit Care. 2023 Dec 6;3:51. doi: 10.1186/s44158-023-00137-z (PMC10702064; doi:10.1186/s44158-023-00137-z)
Supplement: Supplementary file 1 — Additional file 1: Supplementary material 1. Scientific societies provided data. [file 44158_2023_137_MOESM1_ESM.docx]

**Examining Gender Bias in Regional Anesthesia Academic Publishing: A 50-Year Bibliometric Analysis of Regional Anesthesia & Pain Medicine**

**Supplementary Digital Content 1**

| **Year** | **ESRA** | | | | **ASRA** | | | |
| --- | --- | --- | --- | --- | --- | --- | --- | --- |
|  | **Women (n)** | **Men (n)** | **Women (%)** | **Not known (n)** | **Women (n)** | **Men (n)** | **Women (%)** | **Not known (n)** |
| 2008 | 291 | 759 | 27.7 | 889 |  |  |  |  |
| 2009 | 390 | 1028 | 27.5 | 942 |  |  |  |  |
| 2010 | 449 | 1204 | 27.2 | 864 |  |  |  |  |
| 2011 | 521 | 1367 | 27.6 | 642 |  |  |  |  |
| 2012 | 645 | 1614 | 28.6 | 134 |  |  |  |  |
| 2013 | 738 | 1808 | 29.0 | 24 |  |  |  |  |
| 2014 | 807 | 1870 | 30.1 | 32 |  |  |  |  |
| 2015 | 838 | 1950 | 30.1 | 32 |  |  |  |  |
| 2016 | 1050 | 2163 | 32.7 | 470 |  |  |  |  |
| 2017 | 1168 | 2352 | 33.2 | 567 |  |  |  |  |
| 2018 | 1532 | 2682 | 36.4 | 657 |  |  |  |  |
| 2019 | 1814 | 3006 | 37.6 | 757 | 917 | 2755 | 33.3 | 1892 |
| 2020 | 1875 | 3126 | 37.5 | 948 | 830 | 2460 | 33.7 | 1772 |
| 2021 | 1967 | 3192 | 38.1 | 900 | 900 | 2456 | 36.6 | 1807 |
| 2022 | 2184 | 3301 | 39.8 | 917 | 890 | 2411 | 36.9 | 1777 |
| 2023 | 2528 | 3432 | 42.4 | 894 |  |  |  |  |

n:numbers
