# Supplementary material for: Examining gender bias in regional anesthesia academic publishing: a 50-year bibliometric analysis
Source: J Anesth Analg Crit Care. 2023 Dec 6;3:51. doi: 10.1186/s44158-023-00137-z (PMC10702064; doi:10.1186/s44158-023-00137-z)
Supplement: Supplementary file 2 — Additional file 2: Supplementary material 2. Comparison first/last authors and scientific societies composition. [file 44158_2023_137_MOESM2_ESM.docx]

**Examining Gender Bias in Regional Anesthesia Academic Publishing: A 50-Year Bibliometric Analysis of Regional Anesthesia & Pain Medicine**

**Supplementary Digital Content 2**

| Year | Scientific Societies (cumulative) | | | First author | | | | Last author | | | |
| --- | --- | --- | --- | --- | --- | --- | --- | --- | --- | --- | --- |
|  | Women (n) | Women (%) | Men (n) | Women (n) | Women (%) | Men (n) | p-value | Women (n) | Women (%) | Men (n) | p-value |
| 2009 | 291 | 27.7 | 759 | 10 | 28.6 | 25 | 0.911 | 6 | 18.2 | 27 | 0.226 |
| 2009 | 390 | 27.5 | 1028 | 14 | 31.1 | 31 | 0.594 | 4 | 9.3 | 39 | **0.008*** |
| 2010 | 449 | 27.2 | 1204 | 10 | 24.4 | 31 | 0.693 | 2 | 4.7 | 41 | **<0.001*** |
| 2011 | 521 | 27.6 | 1367 | 15 | 26.3 | 42 | 0.831 | 11 | 18.6 | 48 | 0.129 |
| 2012 | 645 | 28.6 | 1614 | 13 | 24.1 | 41 | 0.471 | 8 | 14.5 | 47 | **0.025*** |
| 2013 | 738 | 29.0 | 1808 | 14 | 31.8 | 30 | 0.682 | 7 | 16.7 | 35 | **0.08*** |
| 2014 | 807 | 30.1 | 1870 | 8 | 16.3 | 41 | **0.036*** | 7 | 15.2 | 39 | **0.028*** |
| 2015 | 838 | 30.1 | 1950 | 17 | 32.1 | 36 | 0.751 | 8 | 15.4 | 44 | **0.021*** |
| 2016 | 1050 | 32.7 | 2163 | 19 | 31.1 | 42 | 0.800 | 12 | 20.3 | 47 | **0.045*** |
| 2017 | 1168 | 33.2 | 2352 | 20 | 37.0 | 34 | 0.550 | 11 | 20.8 | 42 | 0.056 |
| 2018 | 1532 | 36.4 | 2682 | 18 | 30.0 | 42 | 0.309 | 9 | 14.5 | 53 | **<0.001*** |
| 2019 | 2731 | 32.2 | 5761 | 22 | 21.4 | 81 | **0.019*** | 20 | 20.0 | 80 | **0.009*** |
| 2020 | 2705 | 32.6 | 5586 | 32 | 41.0 | 46 | 0.115 | 21 | 27.3 | 56 | 0.318 |
| 2021 | 2867 | 33.7 | 5648 | 26 | 39.4 | 40 | 0.327 | 13 | 18.6 | 57 | **<0.001*** |
| 2022 | 3074 | 35.0 | 5712 | 20 | 32.8 | 41 | 0.719 | 13 | 20.6 | 50 | **0.017*** |
| 2023 | 2528 | 42.4 | 3432 | 7 | 29.2 | 17 | 0.189 | 6 | 24.0 | 19 | 0.062 |

* Statistically significant
